# Variation in *Bordetella pertussis* Susceptibility to Erythromycin and Virulence-Related Genotype Changes in China (1970-2014)

**DOI:** 10.1371/journal.pone.0138941

**Published:** 2015-09-25

**Authors:** Ying Yang, Kaihu Yao, Xiang Ma, Wei Shi, Lin Yuan, Yonghong Yang

**Affiliations:** 1 Key Laboratory of Major Diseases in Children and National Key Discipline of Pediatrics (Capital Medical University), Ministry of Education, National Clinical Research Center for Respiratory Diseases, Beijing Key Laboratory of Pediatric Respiratory Infection Diseases, Beijing Pediatric Research Institute, Beijing Children’s Hospital, Capital Medical University, Beijing, China; 2 Respiratory department, Qilu Children’s Hospital, Shandong University, Jinan, China; Universidad Nacional de la Plata, ARGENTINA

## Abstract

**Objectives:**

To investigate changes in virulence-related genotypes and in the antimicrobial susceptibility of *Bordetella pertussis* isolates collected from the 1970s to 2014 in the northern part of China.

**Methods:**

A total of 124 *B*. *pertussis* isolates from three periods, the 1970s, 2000–2008, and May 2013–Sept 2014, were typed by multilocus sequence typing (MLST) and tested for antimicrobial susceptibility and virulence-related genes. A fragment of the 23S rRNA gene from each of the 99 isolates from 2013–2014 was amplified and sequenced.

**Results:**

All isolates from 2000–2008 and 2013–2014 were identified as ST2, whereas isolates from the 1970s were ST1. *PtxA2/ptxC1/ptxP1/prn1/fim2-1/fim3-1/tcfA2*, which was the same as the vaccine strain, was the only type in the 1970s. During the 2000s and 2013–2014, the virulence type *ptxA1/ptxC1/ptxP1/prn1/fim2-1/fim3-1/tcfA2* was dominant, with frequencies of 68.4% and 91.9%, respectively. Nine *ptxP3* strains, which were more virulent, were detected after 2000. All 124 isolates were susceptible to levofloxacin, sulphamethoxazole/trimethoprim and tetracycline. The isolates from the 1970s and 2000–2008 were susceptible to all tested macrolides, whereas 91.9% of the 2013–2014 isolates were highly resistant (minimal inhibitory concentration, MIC >256 μg/ml). No *ptxP3* strain was resistant to macrolides. All erythromycin-resistant strains except for one had the A2047G mutation in the 23S rRNA gene.

**Conclusions:**

Macrolide resistance of the *B*. *pertussis* population has been a serious problem in the northern part of China. Because most of the epidemic clone of the pathogen expresses the same antigen profiles as the vaccine strain, except *ptxA*, improvements in immunization strategies may prevent the spread of infection and drug resistance.

## Introduction

Pertussis, also known as whooping cough, is an infectious disease of the respiratory tract caused mainly by *Bordetella pertussis*. In China, the number of reported cases has dramatically decreased since the introduction of diphtheria-tetanus-pertussis (DTwP) vaccines in the 1980s. However, even with high vaccination rates, many countries have reported increasing pertussis cases, such as the Netherlands, Australia, the U.S., France, and Canada [1–5]. According to the data reported by China CDC posted on The Data-center of China Public Health Science, from the year of 2004 to 2013, the pertussis incidence rate of the whole country were decreased from 0.362 to 0.1264. However, the incidence rate recovered to about 0.2 in 2007 and 2011. Therefore, pertussis is classified as a re-emerging disease. Several causes have been proposed for the resurgence in vaccinated populations, including improved surveillance, development of diagnostic techniques, and waning vaccine-induced immunity. Pathogen adaptation has been considered as the leading cause of waning vaccine-induced immunity[6]. Divergence of major virulence-related antigens such as pertussis toxin (*ptx*), pertactin (*prn*), fimbriae 2 (*Fim2*), *Fim3* and the tracheal colonization factor (*tcfA*) between circulating strains and vaccine strain has been reported[7–9].

Macrolide antibiotics, especially erythromycin, have been used for treatment and prophylaxis of pertussis [10]. Since the first erythromycin-resistant *B*. *pertussis* case was reported in Arizona, USA in 1994[11], other countries have also detected erythromycin-resistant *B*. *pertussis* isolates, including France, Iran and China[10,12,13], It was reported that the mechanism of erythromycin resistance was mainly caused by the A2047G mutation in domain V of 23S rRNA of *B*. *Pertussis* [10,12–14].

Here, we investigated the potential changes in clones, virulence-associated gene patterns, antimicrobial susceptibility, and erythromycin resistance mechanisms of clinical isolates of *B*. *pertussis* collected from the 1970s to 2014 in China.

## Materials and Methods

### Bacterial strains

Overall, 124 *B*. *pertussis* isolates were included in our study. The 124 isolates were collected from three periods: 6 isolates from the 1970s, 19 isolates from 2000–2008 and 99 isolates from May 2013 to September 2014. The 6 *B*. *pertussis* isolates in 1970s were bought from the China National Institute of Control of Pharmaceutical and Biological Products. The remaining 118 clinical *B*. *pertussis* isolates were recovered from nasopharyngeal (NP) swabs of patients or their relatives in Beijing Children’s Hospital (BCH) in China. This facility is the largest children’s hospital in China and currently has more than 10,000 outpatients daily. NP swabs were taken from inpatients and outpatients of the hospital because their clinical symptoms (cough with or without paroxysms, apnea or cyanosis) were suspected of being symptoms of pertussis by their doctors. Personal information including age, sex, address, immunization status, coughing days and antimicrobial usage before swab collection was recorded. A parent and/or legal guardian of each participant signed a written, informed-consent document before enrollment and before any study procedure was performed. This study was reviewed and approved by the Ethics Committee of Beijing Children’s Hospital Affiliated to Capital Medical University. No ethical problems existed in this study.

### Bacterial culture and diagnostic PCR analysis

After collection, NP swabs were immediately spread onto charcoal agar (OXOID, UK) plates and supplemented with 10% defibrinated sheep blood and cephalexin. The plates were incubated in a humidified incubator at 37°C for 3–4 days. Relevant colonies were sub-cultured on a new charcoal blood agar plate without cephalexin. After incubation for 3 days, a potential colony was confirmed by the slide agglutination test with *B*. *pertussis* and *B*. *parapertussis* antisera (Remel Europe Ltd., UK) and also confirmed by polymerase chain reaction (PCR) using IS481 primer [15]. All isolated strains were preserved at -80°C until further analysis.

### Antimicrobial susceptibility test

Antimicrobial susceptibility testing was performed by E-Test and Kirby-Bauer (KB) disk diffusion methods on charcoal agar containing 10% sheep blood [16]. Susceptibility to erythromycin, azithromycin, clarithromycin, clindamycin, levofloxacin, sulphamethoxazole/trimethoprim and tetracycline were tested with the E-test method. Susceptibility to erythromycin and sulphamethoxazole/trimethoprim were also tested with the KB disk diffusion method. The MICs determined by the E-test and inhibition zone sizes determined by disk diffusion were measured after 4 days of incubation. Standardized interpretation criteria currently do not exist for *B*. *pertussis*. According to published studies, inhibition zone sizes of the KB disk diffusion method >42 mm and MICs <64 μg/ml are considered to be susceptible to erythromycin [14, 17,18]. Quality control strains *Haemophilus influenzae* ATCC49247 and *Staphylococcus aureus* ATCC29213 were included in each batch of susceptibility tests.

### Genotyping

The genomic DNA of isolates was extracted using a DNA extraction kit(SBS Genetic Co. Ltd., Beijing, China) following the manufacturer’s instructions. The genes *ptxA*, *ptxC*, *ptxP*, *prn*, *fim2*, *fim3 and tcfA* were amplified and sequenced using previously described procedures. The primers used for amplification of *ptxP* [19], *ptxA* [20], *ptxC* [21], *fim2* [21], *fim3* [22], and *prn* [20,23] were the same as in previous reports. Primers designed for the *tcfA* gene were based on the sequence of the *B*. *pertussis tcfA* gene (GenBank accession no. U16754). The sequences of the primers were 386F: TTGCGCTTGCCCTGCTGGGAG and 995R: GAATCGGACATACCGGAGTCCG. The PCR conditions were as follows: 5 min at 95°C; followed by thirty cycles of 30 s at 95°C, 30 s at 54°C and 90 s at 72°C; and a final extension of 5 min at 72°C.

### Multilocus sequence typing (MLST)

Seven house-keeping genes (*adk*, *fumC*, *glyA*, *tyrB*, *icd*, *pepA* and *pgm*) for multilocus sequence typing (MLST) were amplified and sequenced using recommended primers and conditions [24]. The allele of each gene and the sequence type (ST) based on the alleles of the seven genes were defined according to a public database (http://pubmlst.org/perl).

All PCR amplicons were sequenced at BGI. Sequence information of tested strains was queried to the *Bordetella* MLST sequence-type database.

### Sequencing of the 23S rRNA gene of *B*. *pertussis*

Domain V of 23S rRNA of 99 isolates recovered during May 2013-Sept 2014 were amplified and sequenced using previously described procedures [14]. The DNA sequences were compared to the sequence of the *B*. *pertussis* reference strain Tohama in GenBank (accession no. X68323) (http://www.ncbi.nlm.nih.gov/blast/Blast.cgi).

### Statistical Analysis

Statistical analysis was performed using SPSS version 17.0(SPSS, Chicago, IL). Statistical significance for culture positive rate between antibiotic usage group and no use group was determined by chi-square test.

## Results

### Clinical data on 2013–2014 isolates

Throughout the period from May 2013 to September 2014, 99 isolates were recovered from 274 patients who were suspected of pertussis by the pediatricians in our hospital, with an isolation rate of 36.1%. Among the 274 sampled patients, 228 (83.2%) had already received antibiotics, mostly cephalosporins and/or macrolides. From these 228 patients, 87 (38.2%) isolates were collected. From the remaining 46 patients who either did not take antibiotics or could not provide definitive information, 12 samples cultured positively (26.1%). The two rates were not significantly different (χ^2^ = 2.417, P<0.05).

**Fig 1:**
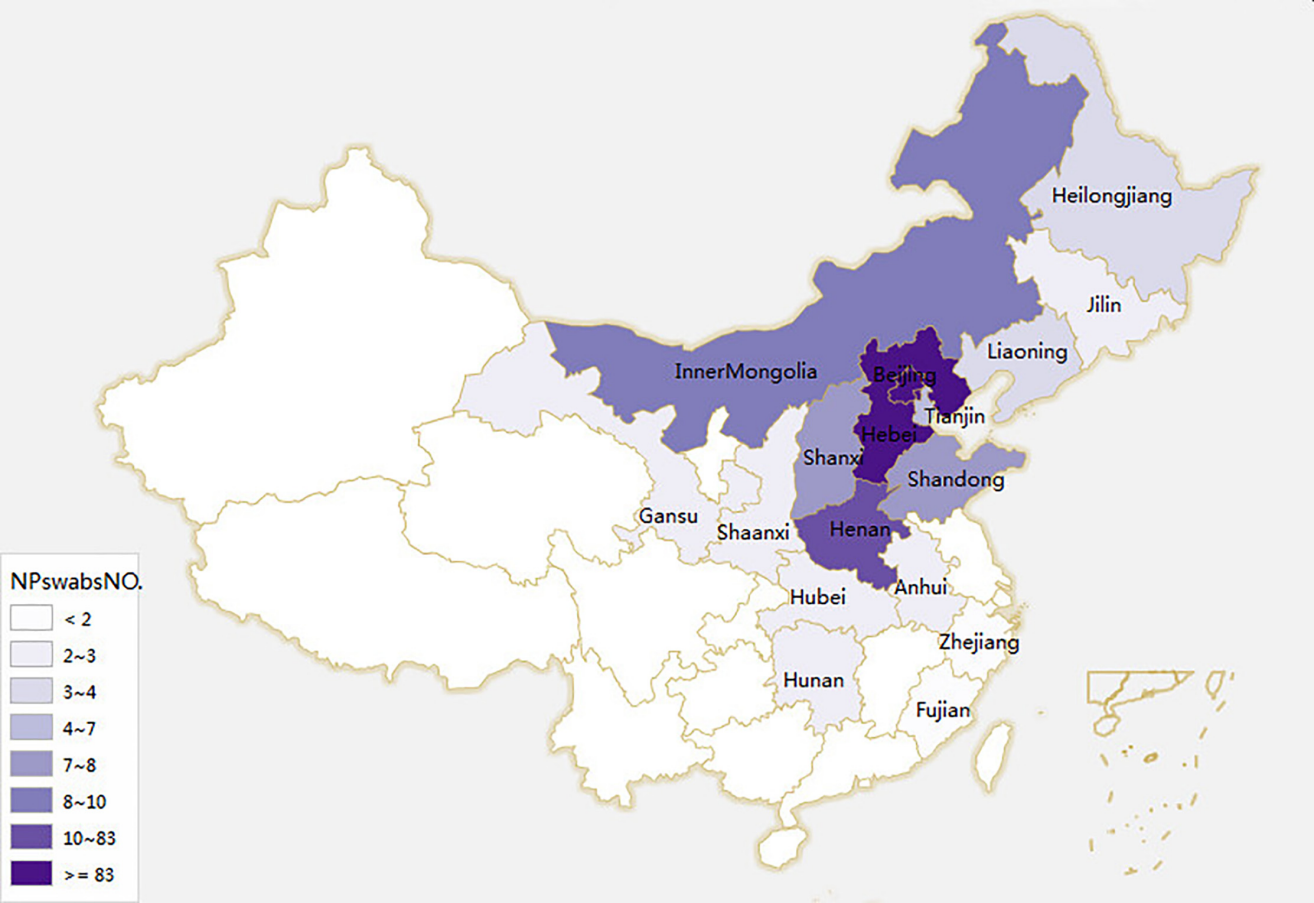
The origins of the 274 patients. Different areas were colored with different shades of color. The deeper the color, the more patients in this area.

The 274 patients were from Beijing (N = 83), Hebei(N = 118), Henan(N = 10), Shandong(N = 7), Shanxi(N = 7), the Inner Mongolia Autonomous Region (N = 8), Tianjin (N = 4), Heilongjiang(N = 3), Jilin(N = 2), Liaoning(N = 3), Shaanxi(N = 2), Gansu(N = 2), Anhui(N = 2), Hunan(N = 2), Hubei(N = 2),. Zhejiang(N = 1) and Fujian(N = 1).The regions of the rest of the patients were not clear. The origins of the 274 patients were shown in a map in Fig 1. From the above-mentioned regions, 36 (43.4%), 46 (39.0%), 4 (40%), 2 (28.6%), 0 (0%), 2 (25%), 1 (25%), 0 (0%), 0 (0%), 1 (33.3%), 1(50%), 0 (0%), 0 (0%), 0 (0%), 2(100%), 0 (0%) and 0 (0%) isolates were collected, respectively.

**Fig 2:**
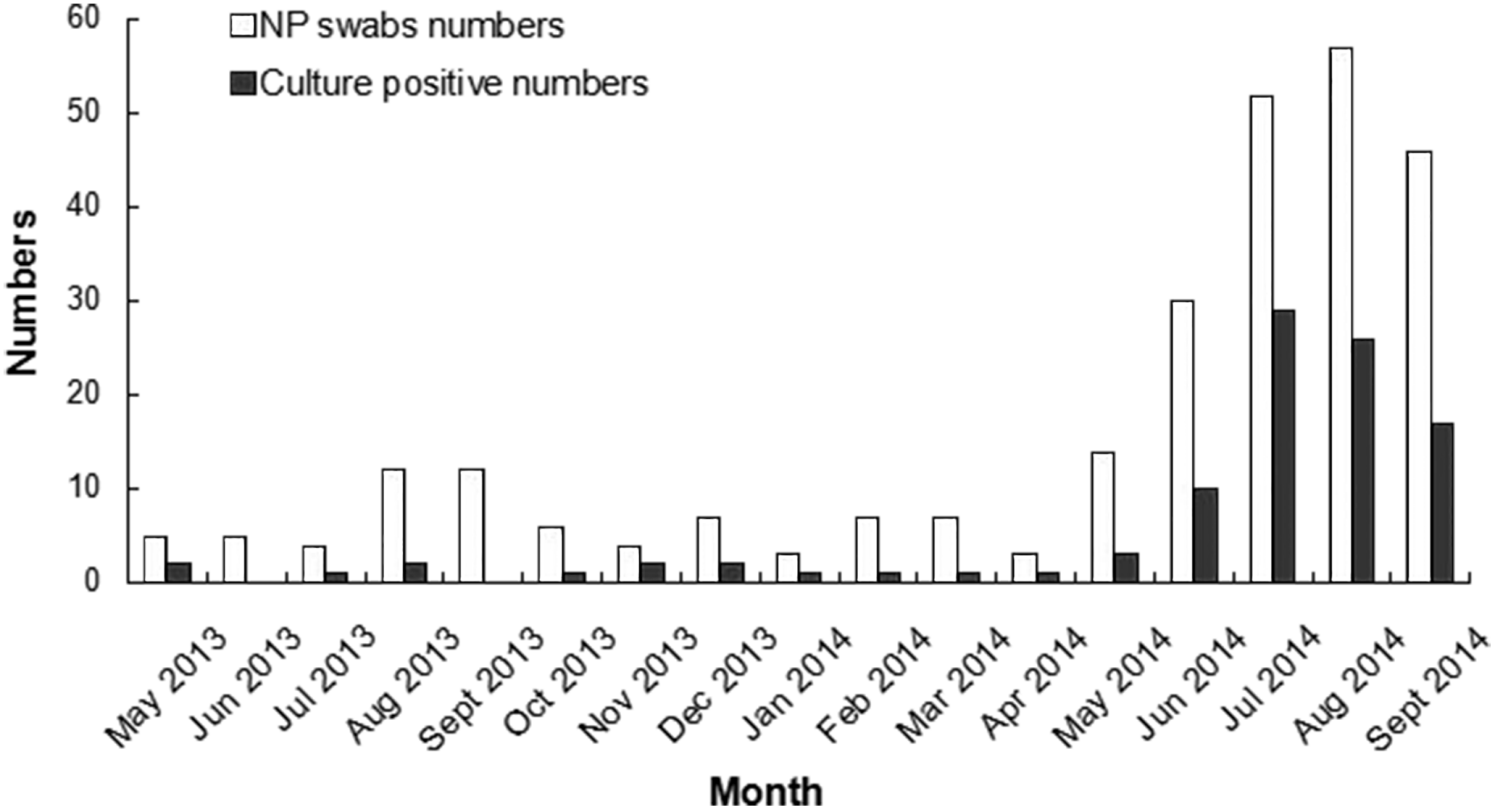
Monthly numbers of NP swabs and culture-positive isolates during May 2013 to Sep 2014.

The monthly numbers of nasopharyngeal swabs(NP) and culture-positive isolates during this period are shown in Fig 2. Although the isolation rate fluctuated significantly during this period, almost every month had confirmed cases. Confirmed cases increased significantly during June-September 2014.

**Fig 3:**
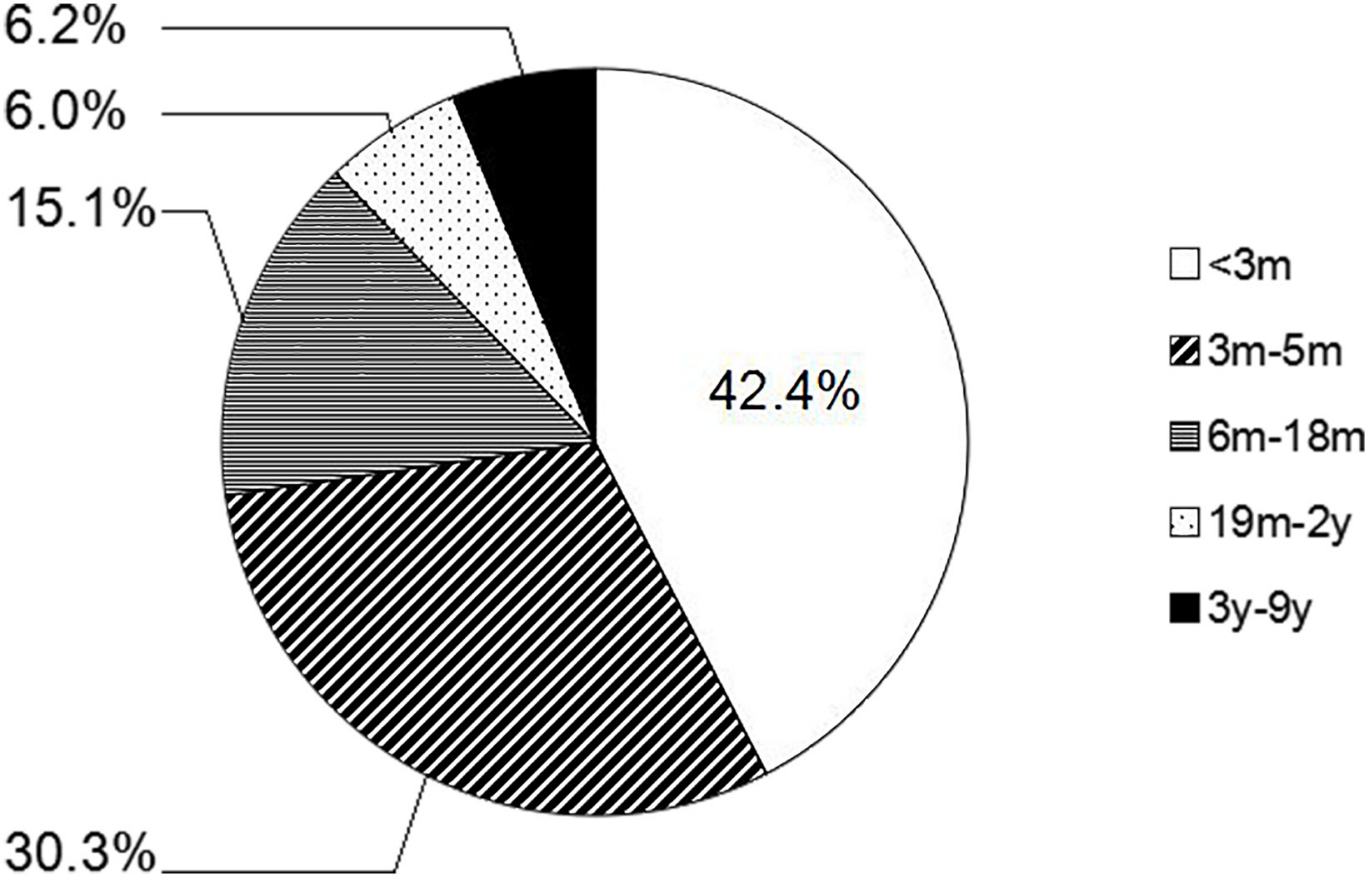
The age distribution of 99 pertussis patients from May 2013 to September 2014.

Among the 274 patients, 116 were younger than 3 months of age, 72 were 3–5 months old, 56 were 6–18 months old, 12 were 19 months to 2 years old, and 18 were 3–12 years old. The isolation rate in each of these age groups was 36.2%, 41.7%, 26.8%, 50.05% and 33.3%, respectively. For the 99 *B*. *pertussis* isolates recovered between May 2013 and September 2014, the proportions of each of the above-mentioned age groups were 42.4%, 30.3%, 15.1%, 6.0% and 6.2%, respectively. The age distribution of 99 *B*. *pertussis*-positive patients sampled between May 2013 and September 2014 is shown in Fig 3.

From the 99 confirmed pertussis patients, 93 patients were recorded with the information of coughing days, 55.9% of who have coughed more than 2 weeks. And 35.5% patients have coughed more than one week but less than two weeks. Eight of the 93 patients have coughed less than one week.

### Antibiotic susceptibility and 23S rRNA gene analysis

Antimicrobial susceptibility test results are shown in Table 1. In the isolates from the 1970s and 2000–2008, the MIC90 for erythromycin, azithromycin, clarithromycin and clindamycin were 0.25/0.125, 0.064/0.064, 2/1, and 2/0.5 (μg/ml), respectively. The inhibition zone diameters of the disk diffusion test of erythromycin were larger than 42 mm (4–55 mm). In 2013–2014, the MIC50 and MIC90 of all the macrolides tested in this study were both >256 μg/ml and 91.9%(91/99) of isolates were tested to be >256 μg/ml of macrolides. The MICs of erythromycin, azithromycin, clarithromycin and clindamycin of the remaining 8 isolates ranged from 0.064–0.125, 0.016–0.047, 0.75–1.5, and 0.016–0.75 μg/ml, respectively. The KB inhibition zone diameters of erythromycin of the 8 isolates were between 45 and 50 mm. In total, the MIC50/MIC90 of levofloxacin, sulphamethoxazole/trimethoprim and tetracycline was 0.75/1, 0.008/0.023 and 1/1.5 μg/ml, respectively, in the three periods. The inhibition zones of sulphamethoxazole/trimethoprim by disk diffusion were between 28 and 65 mm. Additionally, 78.13% of the inhibition zones were >40 mm.

**Table 1:** Antimicrobial susceptibility test results of the 124 *B*. *pertussis* isolates in our study.

	Antibiotic	E-test (μg/ml)			KB disk diffusion (mm)	
		MIC50	MIC90	MIC range	Range of inhibition zone	Rate of susceptibility
Total (N = 124)	erythromycin	>256	>256	0.047->256	6–55	26.6%
	azithromycin	>256	>256	0.008->256	—	—
	clarithromycin	>256	>256	0.75->256	—	—
	clindamycin	>256	>256	0.008->256	—	—
	levofloxacin	0.75	1	0.38–6	—	—
	sulphamethoxazole/ trimethoprim	0.008	0.023	0.001–0.5	28–65	100.0%*
	tetracycline	1	1.5	0.094–16	—	—
1970s (N = 6)	erythromycin	0.094	0.25	0.064–0.25	43 ~ 48	100.0%
	azithromycin	0.032	0.064	0.032–0.064	—	—
	clarithromycin	2	2	1–2	—	—
	clindamycin	1	2	1–2	—	—
	levofloxacin	1	3	0.75–3	—	—
	sulphamethoxazole/ trimethoprim	0.004	0.016	0.001–0.016	35–60	100.0%
	tetracycline	1	16	0.75–16	—	—
2000-2008(N = 19)	erythromycin	0.064	0.125	0.047–0.125	48–55	100.0%
	azithromycin	0.032	0.064	0.032–0.064	—	—
	clarithromycin	1	1	1–1	—	—
	clindamycin	0.5	0.5	0.25–2	—	—
	levofloxacin	1	1	0.5–1	—	—
	sulphamethoxazole/ trimethoprim	0.012	0.094	0.001–0.19	35–65	100.0%
	tetracycline	1	1.5	0.094–1.5	—	—
2013-2014(N = 99)	erythromycin	>256	>256	0.064->256	6–50	8.1%
	azithromycin	>256	>256	0.008->256	—	—
	clarithromycin	>256	>256	0.75->256	—	—
	clindamycin	>256	>256	0.008->256	—	—
	levofloxacin	0.75	1	0.38–6	—	—
	sulphamethoxazole/ trimethoprim	0.008	0.023	0.001–0.5	28–60	100.0%
	tetracycline	1	1.5	0.38–4	—	—

* Reference: The result was referenced to the antimicrobial susceptibility test standard of *Streptococcus pneumoniae* to sulphamethoxazole/trimethoprim.

Ninety strains isolated during May 2013 to September 2014, which showed a >256 μg/ml MIC for macrolides using the E-test method, contained an A2047G mutation in the 23S rRNA gene. No mutation was detected in the remaining nine isolates.

### Genotypes

The virulence-related genotyping results are shown in Table 2. The 6 clinical strains isolated during the 1970s contained *ptxA2/ptxP1/prn1/fim2-1/fim3-1/tcfA2*. In contrast, *ptxA1/ptxC1/ptxP1/prn1/fim2-1/fim3-1/tcfA2* was the major type isolated throughout 2000 to 2008 and May 2013 to September 2014, with frequencies of 68.4% (13/19) and 91.9% (91/99), respectively. In the three periods, only a single genotype for *fim2-1* and *tcfA2* was observed, i.e., *fim2-1* and *tcfA*.

**Table 2:** Genotype profiles of 124 *B*. *pertussis* isolates in Beijing, China.

Genotype profiles	1970s (N = 6)	2000–2008 (N = 19)	2013–2014 (N = 99)	Total (N = 124)
***ptxA2*** */ptxC1/ptxP1/prn1/fim2-1/fim3-1/tcfA2* *	6 (100%)	1 (5.3%)	0	7(5.6%)
***ptxA1*** */ptxC1/ptxP1/prn1/fim2-1/fim3-1/tcfA2*	0	13 (68.4%)	91 (91.9%)	104 (83.9%)
*ptxA1/ptxC1/ptxP1/prn1/fim2-1* */****fim3-4*** */tcfA2*	0	1 (5.3%)	0	1 (0.8%)
*ptxA1/* ***ptxC2*** */* ***ptxP3*** */* ***prn2*** */fim2-1/* ***fim3-2*** */tcfA2*	0	4 (21.0%)	0	4 (3.2%)
*ptxA1/ptxC1/ptxP1/prn1/fim2-1* */****fim3-2*** */tcfA2*	0	0	2 (2.0%)	2 (1.6%)
*ptxA1/* ***ptxC2*** */* ***ptxP3*** */* ***prn2*** */fim2-1/* ***fim3-1*** */tcfA2*	0	0	5 (5.0%)	5 (4.1%)
*ptxA1/ptxC1/ptxP1/* ***prn3*** */fim2-1* */*fim3-1*/tcfA2*	0	0	1 (1.1%)	1 (0.8%)

* According to its' published genome data (CP002695), another China vaccine strain *B*. *pertussis* CS was identified the same genotype profile and ST with the present tested strain 58003.

Three alleles of *fim3* were detected: *fim3-1*, *fim3-2* and *fim3-4*. *Fim3-1* was the major type, with a proportion of 100%, 73.7% and 98.0% in the 1970s, 2000–2008 and 2013–2014, respectively. Between 2000 and 2008, a single *fim3-4* harboring isolate and four *fim3-2* isolates were recovered. In 2013–2014, only two *fim3-2* isolates characterized by *ptxC1/ptxP1/prn1* were detected.

For the *prn* gene, 3 alleles (*prn1*, *prn2*, and *prn3*) were detected among the isolates. In the three periods, *prn1* dominated, with frequencies of 100%, 78.9% and 93.9% in the 1970s, 2000–2008 and 2013–2014, respectively. A single *prn3* isolate was discovered only in the 2013–2014 group.

For alleles of *ptxA*, all isolates from the 1970s harbored *ptxA2*, whereas *ptxA1* predominated in 2000–2008 and 2013–2014, with frequencies of 94.7% and 100%, respectively. These results showed an apparent shift in the predominant allele of *ptxA*.

Two *ptxP* alleles were found in this study, *ptxP1* and *ptxP3*. *PtxP1* accounted for 100%(6/6), 78.9% (15/19) and 94.9% (94/99) in the 1970s, 2000–2008 and 2013–2014, respectively. Four and five *ptxP3* isolates were detected among isolates from 2000–2008 and 2013–2014, respectively, which were combined with *ptxA1/ptxC2/prn2*, along with *fim3-2* in 2000–2008 and *fim3-1* in 2013–2014. The other 2000–2008 strains contained the *ptxP1* allele, all of which were combined with *ptxC1/prn1/fim3-1*, with the exception of a single isolate that combined with *fim3-4*.

### MLST

Two STs, ST1 and ST2, were identified by MLST analysis in this study. These two STs differed by only one gene, i.e., *tyrB*. ST1 contained allele 1, and ST2 contained allele 3. The vaccine strain was identified as ST1 as were most of the 1970s isolates. Two 1970s isolates could not be assigned to STs because no *adk* and *pgm* sequence could be obtained despite several attempts. The two isolates harbored *tyrB* allele 3. All 2000s and 2013–2014 isolates were identified as ST2.

## Discussion

In China, pertussis primary immunization using the whole cell pertussis vaccine (WPV) was introduced in the 1960s. Since 2013, the acellular pertussis vaccine (APV) had fully replaced the use of WPV throughout the country [25]. The immunization program recommended three doses of diphtheria, tetanus and acellular pertussis (DTaP) vaccine to be given at 3, 4 and 5 months of age and a booster dose given at 18–24 months of age. According to official estimates, since 2002 the vaccination coverage rate of the primary three doses of the DTP vaccine has been more than 90% [26]. In 2011, the vaccination coverage of four doses was over 99% [27]. In China, people are not accustomed to carrying vaccination records when visiting doctors. Therefore, the immunization information of some patients included in this study is incomplete. From May 2013 to September 2014, more than 70% of confirmed pertussis patients included in this study were less than 5 months of age, although a small proportion of adolescents were also included. Perhaps because of the wide general usage of antibiotics, no significant difference was found in the isolation rates between the group previously treated with antibiotics and the group with unknown treatment. Based on the regional distribution of our patients, our data are particularly relevant to the northern part of China. In China, it was previously reported that pertussis commonly occurs in spring and summer, with the highest incidences from April through June [28, 29]. The reason for the June-September peak in 2004 in our study is unknown.

The *B*. *pertussis* isolates from the 1970s and 2000–2008 were susceptive to macrolides. In contrast, 91.9% (91/99) of the 2013–2014 isolates were resistant to macrolides (MIC >256 μg/ml). Accordingly, all erythromycin-resistant strains except for one contained the A2047G mutation in the 23S rRNA gene. The erythromycin resistance mechanism of that strain remains to be determined.

Our results confirm that antimicrobial susceptibility of circulating strains in the northern part of China have changed over time, from susceptible to resistant to macrolides. The high resistance rate identified in our study is striking. Although several *B*. *pertussis* isolates have been reported to be resistant to erythromycin in the U.S., France and Iran [12–14], the resistant rates were very low. Additionally, *B*. *pertussis* isolates reported in many other countries are still sensitive to erythromycin, such as United Kingdom, Australia, Japan and Taiwan [30–33]. Conceivably, the reason for the high macrolide resistant rate observed in the present study is antibiotics abuse. In China, azithromycin is the most commonly used antibiotic [34]. Although a high erythromycin-resistance rate of *B*. *pertussis* has been reported in Xi’an, China, erythromycin is still the primary choice for children for clinical antimicrobial treatment in our hospital. Clinicians in our hospital felt that suspected pertussis patients do not respond to erythromycin treatment as well as they did in previous years. But there is no evidence showing that macrolides are useless for the patients infected with *B*. *pertussis*. It was recently reported that *B*. *pertussis* is susceptible to ceftazidime, cefepime and a carbapenem, namely, imipenem [35], which might be used as alternatives for pertussis treatment. Although *B*. *pertussis* was also found to be susceptible to sulphamethoxazole/trimethoprim, levofloxacin and tetracycline, these are not suitable for children.

Major changes also occurred in the circulating strains. Although the 1970s isolates and the vaccine strain were ST1, our MLST analysis showed that all the 2000s and 2013–2014 isolates were ST2. This change came from the change of tyrB allele, from tyrB-1 to tyrB-3 and the change may be driven by the vaccine. In Korea, three ST types (1, 2 and 24) were observed, accounting for 92.6% for ST2, 4.9% for ST1, and 2.5% for ST24. The different STs resulted from the adk and tyrB alleles. ST1 contained adk-1 /tyrB-1, ST2 contained adk-1 /tyrB-3 and ST24 contained adk-2 /tyrB-1. Circulating isolates were ST1 in 1968–1975 and were ST2 in 1999–2009 [36]. Therefore, genotypes of seven housekeeping genes showed similar patterns with Korean isolates.

Although the STs of circulating strains changed over time, most of the genotypes of virulence-related genes remained almost the same as the vaccine strain except ptxA, a finding that differs from those in other countries. In Europe, except for Poland, all circulating isolates carry the non-vaccine type allele *ptxA1*, and non-vaccine type *ptxP3* and *prn2* on average account for 72% and 83%, respectively, of isolates [9]. Furthermore, their genotypes were more diverse than those of isolates in China. In Japan, non-vaccine type *ptxP3* and *prn2* accounted for more than 50% of isolates [37], suggesting that a new vaccine strain or new type of antigen should be included in the vaccine for it to remain effective. In contrast, our results suggest that the vaccine currently in use still covers most of the circulating genotypes. However, there is a need for optimizing the immunization program. Most children are infected by relatives close to them, especially adolescents and adults who have atypical symptoms. Thus, booster doses should be provided to adolescents, adults and the elderly. Additionally, in recent years, immunization of pregnant women has been suggested, which can directly protect them against vaccine-preventable infections as well as protect the fetus and infant via specific antibodies transferred from the mother during pregnancy [38]. In 2013–2014, 91.9% of circulating strains, which contained the same genotype as the vaccine strain except ptxA, were resistant to macrolides. Thus, improved control of the disease may offer additional benefits for the control of antimicrobial resistance in China.

The combination of *ptxC2*, *ptxP3* and *prn2* was found with *fim3-2* in 2000–2008 but with *fim3-1* in 2013–2014. The reasons for this change are unclear. Notably, a number of new alleles such as *ptxP3*, *prn2*, *prn3*, and *fim3-4* were detected in our study, but the proportions of these alleles were very small. Additionally, all of these strains were sensitive to macrolides and did not contain the A2047G mutation in the 23S rRNA gene. This result could explain the observed genotype differences, but the resistance rate in Europe and other regions was found to be very low, indicating that individual strains had varying degrees of resistance and spread.

## Limitations

Our research was not a prospective study. We aimed to obtain as many strains as possible. Thus, we did not put many limits on the inclusion criteria. Nonetheless, the strain numbers in the 1970s and 2000–2008 were small. However, compared to the data reported by Zhang et al. [39], our data are representative of the Chinese population. Additionally, the three time periods of pertussis data in our study are not contiguous. However, we will continuously monitor pertussis cases in the future.
